# Experimental Warming Changes Phenology and Shortens Growing Season of the Dominant Invasive Plant *Bromus tectorum* (Cheatgrass)

**DOI:** 10.3389/fpls.2020.570001

**Published:** 2020-10-15

**Authors:** Armin Howell, Daniel E. Winkler, Michala L. Phillips, Brandon McNellis, Sasha C. Reed

**Affiliations:** ^1^U.S. Geological Survey, Southwest Biological Science Center, Moab, UT, United States; ^2^Department of Forest, Rangeland and Fire Sciences, University of Idaho, Moscow, ID, United States

**Keywords:** *Bromus tectorum*, climate change, dryland, invasive plants, phenology, phenophase, soil moisture, soil temperature

## Abstract

*Bromus tectorum* (cheatgrass) has successfully invaded and established throughout the western United States. *Bromus tectorum* grows early in the season and this early growth allows *B. tectorum* to outcompete native species, which has led to dramatic shifts in ecosystem function and plant community composition after *B. tectorum* invades. If the phenology of native species is unable to track changing climate as effectively as *B. tectorum*’s phenology then climate change may facilitate further invasion. To better understand how *B. tectorum* phenology will respond to future climate, we tracked the timing of *B. tectorum* germination, flowering, and senescence over a decade in three *in situ* climate manipulation experiments with treatments that increased temperatures (2°C and 4°C above ambient), altered precipitation regimes, or applied a combination of each. Linear mixed-effects models were used to analyze treatment effects on the timing of germination, flowering, senescence, and on the length of the vegetative growing season (time from germination to flowering) in each experiment. Altered precipitation treatments were only applied in early years of the study and neither precipitation treatments nor the treatments’ legacies significantly affected *B. tectorum* phenology. The timing of germination did not significantly vary between any warming treatments and their respective ambient plots. However, plots that were warmed had advances in the timing of *B. tectorum* flowering and senescence, as well as shorter vegetative growing seasons. The phenological advances caused by warming increased with increasing degrees of experimental warming. The greatest differences between warmed and ambient plots were seen in the length of the vegetative growing season, which was shortened by approximately 12 and 7 days in the +4°C and +2°C warming levels, respectively. The effects of experimental warming were small compared to the effects of interannual climate variation, suggesting that interactive controls and the timing of multiple climatic factors are important in determining *B. tectorum* phenology. Taken together, these results help elucidate how *B. tectorum* phenology may respond to future climate, increasing our predictive capacity for estimating when to time *B. tectorum* control efforts and how to more effectively manage this exotic annual grass.

## Introduction

Plant community structure and function are governed in part by the timing of key life cycle events (i.e., phenology; Rathcke and Lacey, 1985; Chuine and Beaubien, 2001; Wolkovich and Cleland, 2011; Godoy and Levine, 2014). For example, plant phenology regulates growing season length, which determines the timing of seed production and dispersal, and can ultimately influence the competitive and facultative interactions among plants (e.g., Moeller, 2004). Phenological timing in plants is largely cued to climate conditions, particularly temperature and precipitation (Cleland et al., 2007). Temperature has been linked to the timing of phenophases for numerous systems and across multiple plant growth forms (Partanen et al., 1998; Sherry et al., 2007; Hülber et al., 2010; Wolkovich et al., 2012). Similarly, precipitation patterns are an important driver of plant phenology. Soil moisture, in particular, can stimulate germination and senescence can be delayed in wetter soils (Link et al., 1990; Adondakis and Venable, 2004; Estiarte and Peñuelas, 2015). In drylands, the timing and magnitude of precipitation events are important for determining growing season phenophases, including flowering, seed production, and senescence (Beatley, 1974; Prevéy and Seastedt, 2014). However, the effects of precipitation patterns on phenology are difficult to decipher because precipitation is more variable than, and oftentimes interacts with, temperature (Cleland et al., 2007). Due to these strong climatic controls over phenology, climate change is having large effects on the timing of green up, flowering, and senescence for plants in drylands and around the world (Kimball et al., 2010; Wolkovich et al., 2012).

Future climate scenarios for most drylands predict an increased severity and frequency of drought, greater variability in precipitation, and warmer temperatures (Cayan et al., 2010; IPCC, 2014; Reidmiller et al., 2018). Additionally, warmer temperatures will likely change the form of precipitation, as cooler months will experience more rain instead of snow (Barnett et al., 2005). Such changes to climate could alter species ranges, create new temporal niches in plant communities, and differentially affect multiple plant species. For example, winter annuals that grow early in the season are often limited by cold winter temperatures and may be more likely to germinate in response to winter rains resulting from increased temperatures. This earlier germination could infer a competitive advantage over plants that initiate growth later in the season, provided the temperatures are sufficiently high to meet germination requirements, but not too low to induce mortality of the seedlings (Beatley, 1974; Kimball et al., 2010; Wolkovich and Cleland, 2011). Additionally, invasive annual plants often employ more flexible resource acquisition strategies that can facilitate rapid phenological responses (Funk, 2013; Wolkovich and Cleland, 2014; Winkler et al., 2018). Given this, invasive plants may be better adapted to tolerate climate change than co-occurring native annuals and perennials, especially those existing at range edges or experiencing novel climate regimes (Seastedt et al., 2008; Willis et al., 2010; Wolkovich and Cleland, 2014; Ashbacher and Cleland, 2015; Phillips, 2019). Further, invasive annual plants may germinate rapidly, increase growth rates, or advance flower and seed production in response to earlier spring temperatures, unseasonably early rains, and nutrient pulses (Esque et al., 2010; Willis et al., 2010). Often, invasive plants are better suited to exploit temporal niches than native plants and this can allow invasives to exploit resources at times when natives are inactive, which can lead to subsequent shifts in community composition, vegetation cover, nutrient cycling, and fire regimes (D’Antonio and Vitousek, 1992; Bradley et al., 2010; Willis et al., 2010; Dickens and Allen, 2014). Together, these traits that allow rapid concerted responses to changing climate have led to predictions of increased invasive species’ ranges and abundance under future climate scenarios (Bradley et al., 2010), however, data assessing climate change effects on the timing and success of exotic plants are relatively rare.

*Bromus tectorum* (cheatgrass) is one of the most destructive invasive annual plants in North American dryland ecosystems (Knapp, 1996). Areas where *B. tectorum* has naturalized have seen subsequent alterations to fire regimes, native plant composition and survival, and hydrological and nutrient cycles (D’Antonio and Vitousek, 1992; Rimer and Evans, 2006; Keeley and Brennan, 2012). *Bromus tectorum* is a native of central Eurasia and was introduced to the western United States in the late 1800s: the exotic grass is highly invasive, and it spread rapidly across the West. This species now covers an estimated 22.7 million hectares in the United States (Duncan et al., 2004) and an estimated one-third of the Great Basin Desert (Bradley et al., 2018). Invaded areas include at least 20 million hectares where *B. tectoru*m is dominant, or nearly so (Novak and Mack, 2001). Additionally, *B. tectorum* is continuing to spread at an estimated rate of 14% (Duncan et al., 2004), moving into high elevation areas after disturbance (Mealor et al., 2012), with millions of hectares considered highly likely to experience future invasion (Pellant and Hall, 1994). It has been suggested that *B. tectoru*m is able to successfully invade and establish because it has more competitive life history traits than native plants, traits that include high specific leaf area, high nitrogen-use efficiency, faster relative growth rates, and higher and faster rates of seed production (Hulbert, 1955; Harris, 1967; Wainwright et al., 2012). Using these traits, *B. tectorum* has been shown to respond to disturbances including fire, nutrient deposition, and climate change via an increased capacity to tune growth strategies (including phenology) to extant conditions, thereby increasing invasiveness (He et al., 2011; Liu et al., 2013; Peeler and Smithwick, 2018; Mesa and Dlugosch, 2020; Williamson et al., 2020). Once established, *B. tectorum* can limit water availability and the species’ relatively early phenology can further exacerbate negative impacts on native plants (Harris, 1967; Melgoza et al., 1990; Eliason and Allen, 1997; Booth et al., 2003). *Bromus tectorum’s* early and flexible phenology may synergize with future climate conditions and increase its competitive advantage over native plants, as *B. tectorum* may be able to take advantage of earlier growing seasons and early season rain events that native plants cannot utilize (Abatzoglou and Kolden, 2011; Bradley et al., 2016).

Understanding how climate change will affect *B. tectorum* phenology would further our ability to identify locations that are more susceptible to invasion, including identification of invasion hotspots that could benefit from increased control efforts (Bishop et al., 2019). Interpreting the climate controls over *B. tectorum* phenology would also improve spatial-temporal forecasts of how *B. tectorum* abundance and life cycle timing affect changes in competitiveness and fuel loads into the future (Compagnoni and Adler, 2014; Underwood et al., 2019). Further, successful control of *B. tectorum* and promotion of native vegetation is contingent upon *B. tectorum* eradication *prior* to seed production and before native plants become active (Lehnhoff et al., 2019). Accordingly, successful management relies on predicting the timing of *B. tectorum* phenophases and how invasion will be shaped by future climate (Garbowski et al., 2019). However, in spite of the utility of an improved understanding of *B. tectorum* phenological responses to climate change and of the many projections of potential effects on *B. tectorum* success, our knowledge of the controls and magnitude of these effects on *B. tectorum* phenology remains limited. This relatively poor understanding stems from both a lack of long-term observations of *B. tectorum* phenology, and from a low number of climate manipulation experiments in the ecosystems where *B. tectorum* exists (Aronson and McNulty, 2009).

To address this important unknown, we followed three stages of *B. tectorum* phenology for a decade in *in situ* climate manipulation experiments at two sites on the Colorado Plateau, United States. We used infrared lamps to actively warm plants and soils and hand watering to alter precipitation regimes in a full factorial design. We tracked the phenology of *B. tectorum* germination, flowering, and senescence weekly throughout the growing season to test the following hypotheses: 1. Warming treatments will advance *B. tectorum* germination, flowering, and senescence and will shorten the length of the vegetative growing season. 2. altered precipitation or the legacy of altered precipitation will delay phenology and extend *B. tectorum*‘s growing season, and 3. background climate conditions will determine the strength of treatment effects (i.e., the magnitude of treatment effects will vary by year depending on that year’s weather). Our research builds upon a previous study that assessed the first three years of *B. tectorum* phenology under ambient and warmed conditions (Zelikova et al., 2013) and allows us to explore whether treatment patterns persisted throughout a decade, as well as to elucidate how interannual variability controls this invasive species’ phenological patterns.

## Materials and Methods

### Study Location

*Bromus tectorum* phenology was assessed in three complementary climate manipulation experiments, which have been described previously (Reed et al., 2012; Zelikova et al., 2012, 2013; Wertin et al., 2015, 2017; Winkler et al., 2019). The three studies were set up in two cool desert ecosystems of the Colorado Plateau, United States. Two of the experiments were installed at the same site near Castle Valley, Utah (36.675 N, -109.416W, 1310 m elevation). The soils at the Castle Valley site are classified as sandy loam, calcareous, Rizno series. Soil texture at this site is 61% sand, 25% silt, and 14% clay [as assessed by the Soils Lab at Brigham Young University using a hydrometer (Day, 1965)]. Vegetation cover at the Castle Valley site is dominated by the perennial C_4_ grass *Pleuraphis jamesii*, the perennial C_3_ grass *Achnatherum hymenoides*, the C_4_ shrub *Atriplex confertifolia*, and the invasive annual C_3_ grass *B. tectorum*. Both experiments at the Castle Valley site have a west aspect and the slope ranges from 10°–13°. Prior to establishing the experiments, the Castle Valley site had experienced limited to no anthropogenic activity.

The second site is approximately 35 km from the Castle Valley site and is near Moab, Utah (38.31 N, -109.28 W, 1227 m elevation). Soils at the Moab site are sandy loam, Sheppard series, with a thin petrocalcic layer at a depth of 0.5 m. The soil texture at the site is 92% sand, 2% silt, and 5% clay as assessed by the texture-by-feel method (Salley et al., 2018). The vegetation cover is dominated by the perennial C_3_ grass *Achnatherum hymenoides* and the invasive annual C_3_ grass *B. tectorum*. The Moab site has a slope of 4° with a southern aspect. The site was periodically grazed between 1900 and 2008, and grazing was excluded prior to establishing the experimental plots. Both the Castle Valley and Moab sites have a 1.5 m tall electric fence and 0.5 m tall mesh fence around their perimeters to exclude grazing of cattle and wildlife.

### Climate Manipulation Treatments

The three complementary climate manipulation experiments experienced one of two levels of warming and one of two types of precipitation treatments (Table 1). One experiment at the Castle Valley site began in 2005 and for most years was warmed to 4 °C above ambient: this experiment will be subsequently referred to as “CV4”. The second experiment at the Castle Valley site began in the Fall of 2008 and increased plot temperatures 2 °C above ambient: this experiment will be subsequently referred to as “CV2”. The experiment at the Moab site also began in the Fall of 2008 and increased plot temperatures 2 °C above ambient. This experiment will be subsequently referred to as “M2”.

**TABLE 1:** Experiment names, site location, year treatments began, degree of warming above ambient, and type of altered precipitation treatment are described for each of the three experiments.

Experiment name	Location	Year of initiation	Warming level	Precipitation treatment
CV4	Castle Valley, UT	2005	4 °C	Small frequent
CV2	Castle Valley, UT	2008	2 °C	Large infrequent
M2	Moab, UT	2008	2 °C	None

The 20 plots comprising the CV4 experiment were originally selected to have similar biocrust cover (Zelikova et al., 2012) and were set up in five blocks to account for downslope spatial variation within the site. Within each block, warming, altered precipitation, warming + altered precipitation, and ambient treatments were randomly assigned to each plot in a full factorial design (*n* = 5 plots per treatment for a total of 20 plots). In January 2009, temperature treatments in the CV4 plots were increased from 2°C to 4°C above ambient to better capture the range of expected future temperatures. The CV4 plots were subjected to altered precipitation treatments that were intended to mimic small, frequent monsoonal precipitation events. Altered precipitation treatments began in the CV4 plots in the summer of 2006 and continued until 2012 when they were discontinued due to an opportunity to assess biocrust recovery with and without warming (see Reed et al., 2012 and Zelikova et al., 2012 for additional details). For the CV4 plots, water was applied in 1.2 mm events 5 times every two weeks. Altered precipitation treatments were applied approximately between June 15 and September 15 each year.

In the fall of 2008, the CV2 plots were constructed within the same large enclosure as the CV4 plots and the M2 plots were installed near Moab, Utah. The 20 CV2 plots were selected to have similar vegetation cover and, as with CV4, the treatments—warming, altered precipitation, warming + altered precipitation, and untreated ambient—were randomly assigned to five blocks to account for downslope spatial variation (*n* = 5 plots per treatment for a total of 20 plots). In contrast to the small, frequent monsoonal precipitation treatments in the CV4 experiment, CV2 had a larger, less frequent altered precipitation treatment which was designed to mimic monsoonal events based on a 30-year average (Table 1). Altered precipitation treatments in the CV2 plots began in the summer of 2009 and continued until 2012 when treatments ended because plants showed no responses to the treatment (Wertin et al., 2015). Altered precipitation treatments were applied with sprayers each year between approximately June 15 and September 15. The M2 plots were selected for similar vegetation cover, assigned to five blocks to account for cross-site spatial variation, and plots within each block were randomly assigned warming or control treatments (*n* = 5 plots in each treatment for a total of 10 plots). No watering treatments were applied to the M2 site.

Two Kalglo MRM 2408 infrared heaters (Kalglo Electronics Co., Inc., Bath, PA, United States) were placed 1.3 m above the soil surface to apply warming treatments at each site. We chose infrared heaters to experimentally warm plots because they have been shown to have a high degree of manipulative accuracy and minimally disturb soil surfaces, which is important for our study area where fragile soil surface biota strongly regulate function (Kimball, 2005; Aronson and McNulty, 2009; Reed et al., 2012). All lamps were oriented in a north-south direction to minimize shading and the warming design followed Harte et al. (1995). All plots that did not receive warming treatments were fitted with “dummy” heaters with the same dimensions and orientation. Wertin et al. (2015) describe how experimental temperatures were achieved using soil temperature sensors at 5 cm depths and how increasing the temperatures at these depths by 3.57°C and 1.58°C correspond to elevated soil surface temperatures of 4 °C and 2 °C, respectively. Campbell Scientific CR1000 data loggers (Campbell Scientific Inc., Logan, UT, United States) were programmed to constantly monitor 5 cm deep soil temperatures and toggled lamps on and off in order to maintain 5 cm deep soil temperature differences of +3.57°C in the +4°C warming plots and +1.58°C in the +2°C warming plots relative to their ambient controls. The infrared lamps used for warming have been tested and shown not to emit any visible light that would affect plant phenology (Kimball, 2005).

All plots at each site were rectangles that measured 2.5 × 2 m and were oriented such that the long (2.5 m) side ran east to west. All plots were edged with vinyl plastic flashing to prevent overland water flow and minimize roots from growing into or out of the plots. In both CV experiments, where soils are shallow (Whitney et al., 2017), flashing was buried to a depth of 15 cm. In the M2 experiment, where soils are deeper, the flashing was buried to a depth of 30 cm. At the center of both sites, meteorological stations were installed with 1-min measurement intervals and reported hourly averages. Precipitation was measured with Texas Instruments TE525WS tipping bucket rain gauges (Texas Electronics Inc., Dallas, TX, United States).

In each plot, soil temperature and moisture were measured every 15 min and averaged hourly at three soil depths. Soil microclimate probes were placed at 2, 5, and 10 cm depths in the CV2 and CV4 plots and at 5, 10, and 20 cm in the M2 plots (sensor depths varied between sites due to soil depth differences). For soil temperature, three-tipped thermopiles were constructed from 24-gauge Type-E thermocouple wires (Omega Engineering Inc., Norwalk, CT, United States). To assess soil volumetric water content, a combination of CS616 water content reflectometer and Decagon EC-5 soil moisture probes were used (Campbell Scientific, Logan, UT, United States and Decagon Devices, Pullman, WA, United States). CS616 water content reflectometers were installed at 2 cm depths in all plots at CV2 and CV4 and at 5 cm depths in all plots at the M2 site. EC-5 soil moisture probes were installed at 5 and 10 cm depths in all plots at both CV sites and at depths of 10 and 20 cm in the all plots at the M2 site. Foliar temperatures were measured to determine if plants in the experiments received higher levels of warming than what was recorded in the soils. This was achieved using Apogee SI-121 infrared radiometer sensors placed 15–80 cm above 1 focal plant canopy in randomly assigned warming and ambient plots in both CV experiments, depending on the canopy size (Apogee Instruments, Inc., Logan, UT, United States). Plants in the CV4 and CV2 experiments experienced temperatures that were on average 5 °C and 1.6°C higher in the warmed plots than ambient plots, respectively.

### Phenology Measurements

Phenology measurements began in 2009 at all sites and followed a modified field observation protocol based on Wein and West (1971). Plots were scored based on the timing and duration of germination, flowering, and senescence phenophases and the Julian day when the transition from one phenophase to another occurred was recorded. Transitions from one phenophase to the next were defined as follows: germination was recorded as the first sign of germination in the plot, flowering was recorded as the first sign of flower budburst in the plot, senescence was recorded when all plants had fully senesced in the plot, and vegetative growing season was calculated as the number of days between the first sign of germination and the first sign of flowering in a plot. In all years, phenophases were scored on a weekly basis but the timing of initiation and termination of weekly surveys differed among years. In 2009 and 2011, surveys started in April and February, respectively, and ended when all plants were fully senesced in June. In 2010, surveys began in February and continued for the year and in 2015–2019 surveys occurred weekly throughout the year, thus these years captured all necessary phenological stages. Phenology was only inconsistently measured from 2012–2014. Due to the shortened measurement times in 2009 and the inconsistent measurements in 2012–2014, only data from 2010, 2011, and 2015–2019 were used in this analysis. During fall 2017, *B. tectorum* germinated at the M2 site, but plants senesced shortly after in early winter and new plants germinated the following spring. These germination and senescence events were excluded from statistical analyses since they were outliers that only occurred once during the experiment.

### Statistical Analyses

Mean daily soil volumetric water content was aggregated from hourly measurements that began December 1, 2008 and ended December 12, 2019. Data were collected at 10 cm depth from the 3 Decagon EC-5 probes in each plot (Decagon Devices, Pullman, WA, United States) (*n* = 20, 20, 10 in the CV4, CV2, and M2 experiments, respectively). In order to zero each sensor and account for sensor drift, the minimum volumetric water content value for each sensor in each year was subtracted from all other volumetric water content values for each sensor in that year. In addition, all soil moisture data collected in frozen soils (≤0°C) were discarded due to erratic probe behavior.

Soil moisture data were modeled using linear mixed-effects models using restricted maximum-likelihood (Harrison et al., 2018). A first-order continuous time covariate nested within each experimental plot was included to account for strong temporal autocorrelation between daily soil moisture (Pinheiro and Bates, 2002). Plot effects were in turn nested within blocks, while blocks were nested within year to account for spatial heterogeneity across experiments, as well as strong annual variability in climate. Continuous time covariates were scaled and centered to improve model convergence. Altered precipitation treatments did not significantly affect phenology in our experiments but warming treatments did. Therefore, this analysis of soil moisture focused only on the effects warming treatments in order to explore mechanisms for warming treatment effects on *B. tectorum* phenology. Significance of warming treatment effects was assessed using treatment-group pairwise comparisons of estimated marginal means adjusted for multiple comparisons using Tukey’s method (Lenth, 2016). Separate models with identical terms were constructed for each experiment. All soil moisture data were analyzed using R version 3.6.3 and utilized the packages ‘nlme’ and ‘emmeans’ (Lenth, 2020; Pinheiro et al., 2020; RCore Team, 2020).

*Bromus tectorum* phenological response to warming and watering treatments was analyzed using linear mixed-effects models with a restricted maximum likelihood approach (Zuur et al., 2006; Bolker et al., 2009; Nakagawa and Schielzeth, 2013). Models were designed to test the treatment effects of warming, altered precipitation, and their interaction on the timing of *B. tectorum* phenology. Separate models were constructed for each experiment because sample size and both warming and altered precipitation treatments differed across experiments. All models included random intercepts for the 5 blocks in each experiment to account for spatial variation. Each model also included random intercepts for year, as well as random slopes for warming treatments in each year. This allowed accounting for year-to-year climate variability in *B. tectorum* phenology, as well as examination of the interaction between warming treatment effects and yearly weather. Two models were fitted for each phenology variable (germination, flowering, senescence, and vegetative growing season) at each experiment (CV4, CV2, and M2) for a total of 24 candidate models. The first of the two models had additive fixed effects of warming and altered precipitation and the second model had interacting warming and altered precipitation effects in addition to the additive warming and altered precipitation effects. For each phenology variable at each site, the final models were selected with a likelihood ratio test (Bates, 2010). Twelve final models were selected, one for the timing of germination, flowering, senescence, and total number of vegetative days (time from germination to the initiation of flowering) in each of the 3 experiments. No models with interacting warming and altered precipitation were selected and so treatment effects were tested using Type-II ANOVA on each candidate model (Kuznetsova et al., 2017; Luke, 2017). The contribution of the interaction between each year and warming treatment was quantified using conditional modes of the random effects along with their 95% confidence intervals (Fox et al., 2015). Linear mixed-effects model analysis and ANOVA analysis on the models utilized the ‘lme4’ package (Bates et al., 2015).

We further explored aspects of interannual variation by analyzing relationships of yearly changes in soil moisture and temperature on the timing of *B. tectorum* phenology. Mean annual soil moisture data were collected at 10 cm depth from the 3 Decagon EC-5 probes in each plot (Decagon Devices, Pullman, WA, United States) (*n* = 20, 20, 10 in the CV4, CV2, and M2 experiments, respectively). In order to zero each sensor and account for sensor drift, the minimum volumetric water content value for each sensor in each year was subtracted from all other volumetric water content values for each sensor in that year. In addition, all soil moisture data collected in frozen soils (≤0°C) were discarded due to erratic probe behavior. Mean annual soil temperature data were collected at 10 cm depth from 3 three-tipped thermopiles in each plot. Mean annual soil moisture and temperature were aggregated from hourly measurements in each year when phenology measurements were recorded (2011, 2016–2019 for germination and vegetative growing season and 2010, 2011, 2015–2019 for flowering and senescence).

In total, twelve linear mixed effects models were constructed to explore microclimate effects (soil moisture and temperature) on each of the four phenology variables (germination, flowering, senescence, and vegetative growing season) in each of the three experiments (CV4, CV2, and M2). All models included additive fixed effects of mean annual soil moisture and mean annual soil temperature. Each model included year and block as random intercepts to account for other aspects of yearly variation beyond changes in soil moisture and temperature and spatial variation across each experiment. Marginal r^2^ values were calculated to show how much variation in phenological timing was explained by the fixed effects of the models (10 cm soil moisture and soil temperature). Conditional r^2^ values were calculated to show how much variation in phenological timing was explained by other aspects of annual variation and spatial variation across each experiment. Marginal and conditional r^2^ values were determined with the r.squaredGLMM function in the ‘car’ package (Nakagawa and Schielzeth, 2013; Fox and Weisberg, 2019). The significance of mean annual soil moisture and temperature were determined with Type-II ANOVAs on each of the 12 models (Kuznetsova et al., 2017).

## Results

In the CV4 and CV2 experiments, all final models had additive warming and altered precipitation fixed effects and no final candidate models included warming and altered precipitation treatment interactions. Final models for M2 included warming as the only fixed effect, because no altered precipitation treatments were applied in this experiment. Model results can be found in Table 2.

**TABLE 2:** Results from linear mixed effects models.

Experiment	Phenology variable	Warming effect estimate (DOY)	Standard error	Observations	Degrees of freedom	F value	Warming Pr (>F)
CV4	Germination	3.11	1.52	99	4.01	3.36	0.140
**CV4**	**Flowering**	**−7.78**	**2.01**	**140**	**6.00**	**12.90**	**0.006**
**CV4**	**Senescence**	**−3.03**	**0.98**	**140**	**6.00**	**8.19**	**0.029**
**CV4**	**Vegetative Growing Season**	**−12.12**	**2.19**	**99**	**5.56**	**27.4**	**0.002**
CV2	Germination	2.50	1.50	100	4.00	2.43	0.194
**CV2**	**Flowering**	**−5.86**	**1.35**	**140**	**6.00**	**16.89**	**0.006**
**CV2**	**Senescence**	**−1.03**	**0.44**	**140**	**18.52**	**5.24**	**0.034**
**CV2**	**Vegetative Growing Season**	**−9.24**	**2.37**	**100**	**4.00**	**12.50**	**0.024**
M2	Germination	1.75	2.06	40	7.43	7.30	0.458
**M2**	**Flowering**	**−3.86**	**1.32**	**70**	**7**	**6.22**	**0.035**
**M2**	**Senescence**	**−5.14**	**1.9**	**70**	**7**	**0.64**	**0.047**
**M2**	**Vegetative Growing Season**	**−6.3**	**2.50**	**40**	**29.34**	**6.15**	**0.020**

*Warming effect estimate is the number of days the warming treatments advanced or delayed (+ or – sign, respectively) the timing of *B. tectorum* germination, flowering, senescence, or the length of the vegetative growing season. Negative values show earlier timing of phenophases in the warming relative to ambient plots and positive values show later timing. Degrees of freedom, *F* values, and *p* values were determined from Type-II ANOVAs. Significant (*p* < 0.05) treatment effects on phenophases are shown in bold font.*

**FIGURE 1:**
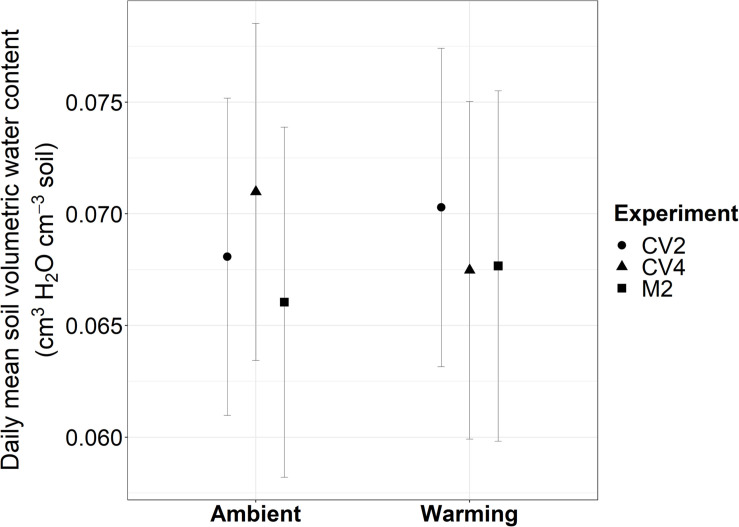
Daily mean soil volumetric water content estimated from marginal means of linear mixed-effects models from 2009–2019 in the CV4, CV2, and M2 experiments. Experiments are represented with different shaped polygons: circles for CV2, triangles for CV4, and squares for M2. Error bars are 95% confidence intervals calculated on marginal means. There were no significant differences in mean soil volumetric water content between warmed and ambient treatments in any of the three experiments.

From 2009 to 2019, experimental warming increased 10 cm deep soil temperatures above ambient soil temperatures an average of 2.73°C in the CV4 plots, 1.34°C in the CV2 plots, and 1.25°C in the M2 plots (Supplementary Figure 1). These increased temperatures are similar yet muted compared with the warming differences observed at 5 cm depths (Zelikova et al., 2013; Wertin et al., 2015, 2017). From 2009–2019 there were no differences in mean volumetric water content between warmed and ambient plots for any of the experiments when averaged across each year (Figure 1). Wertin et al. (2015) describe, in detail, the effects of the altered precipitation treatments on both soil moisture and temperature at 5 cm depths. Briefly, watering treatments did not significantly affect soil temperatures in either the altered precipitation plots or the warming + altered precipitation plots. Additionally, only the small-frequent altered precipitation treatments of the CV4 sites significantly increased soil moisture during the months when plots were watered and the large-frequent watering at the CV2 sites did not show significant measurable effects on soil moisture at the 5 cm measurement depth.

**FIGURE 2:**
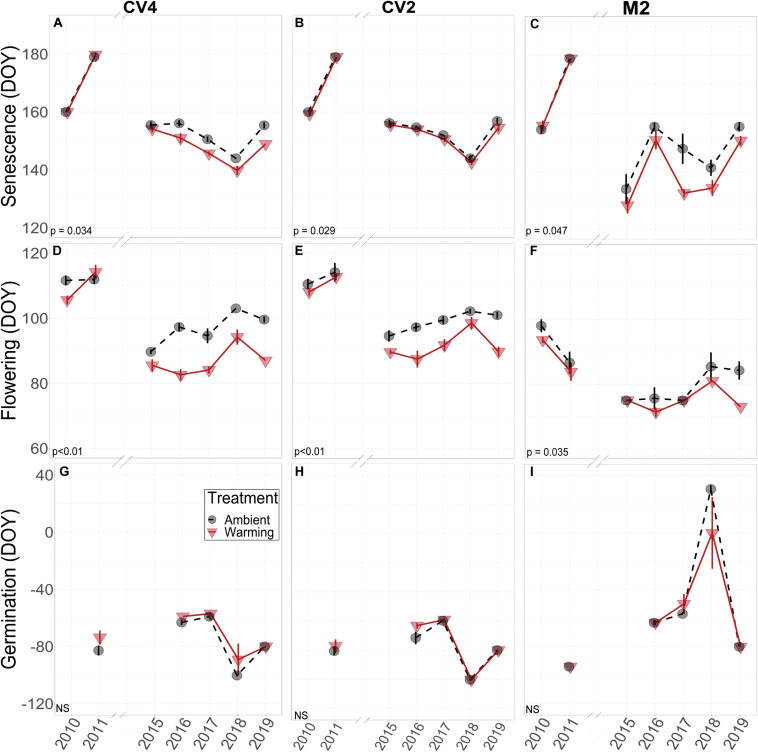
Empirical data for *B. tectorum* senescence **(A–C)**, flowering **(D–F)**, and germination **(G–I)** timing measured weekly in 2010, 2011, and 2015–2019. All phenophases are shown as the average day of year that each phenophase was first observed in each treatment. A value of 0 for day of year corresponds to January 1. A lower value on the y-axis represents earlier timing and a higher value represents later timing. Negative values for day of year indicate that the phenological event took place that number of days prior to January 1. For example, most germination events took place in the Fall and they are shown as negative days for that year (i.e., germination in 2011 took place in the Fall of 2010). Warming treatments are depicted as red triangles and are connected with solid red lines. Ambient treatments are shown with black circles and are connected with dashed black lines. Standard errors of the means are shown with vertical bars associated with the appropriate polygon. Significance of treatment effects is shown in the bottom left of each panel, with NS denoting non-significant effects. Significant differences between the treatment means were determined by Type-II ANOVAs.

**FIGURE 3:**
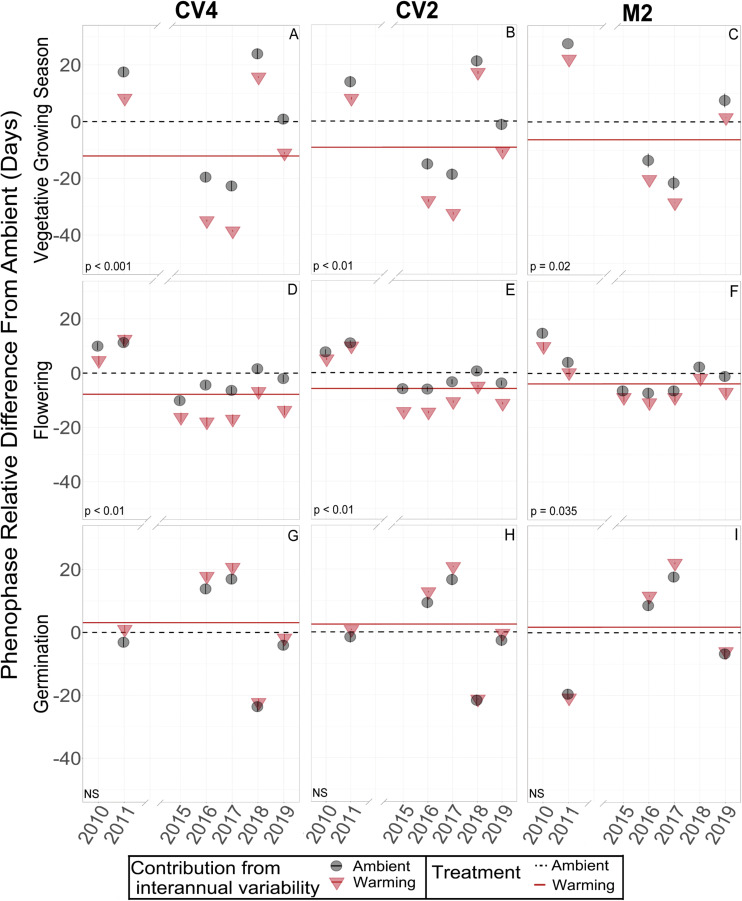
Model decompositions for vegetative growing season **(A–C)**, flowering **(D–F)**, and germination **(G–I)** of *B. tectorum* in each of the three climate manipulation experiments. The estimated mean number of days each phenological stage was delayed or advanced are represented by the horizontal lines. A lower value on the y-axis represents earlier timing and a higher value represents later timing. The estimated means for the ambient plots are represented by the dashed black horizontal lines and are set to zero as all data are shown relative to the ambient plots for each site and each phenological stage. Solid red horizontal lines represent the mean difference in timing (in Julian days) of the warmed plots relative to the ambient plots (black dashed line). When the solid red line is below the dashed black, line there is an estimated warming-induced advance of that phenophase. When the solid red line is above the dashed black line, there is an estimated warming-induced delay of that phenophase. Significance of treatment effects is shown in the bottom left of each panel, with NS denoting non-significant effects. Significant differences between the treatment means were determined by Type-II ANOVAs. The conditional modes of the random effects for the ambient and warming treatments in each year are shown with black circles or red triangles, respectively. Estimated confidence intervals for these conditional modes are shown with vertical bars within the circles or triangles. Interannual variability of these conditional modes are estimated by their difference each year from the horizontal lines of the means.

Across the years, *B. tectorum* phenology did not respond to altered precipitation treatments nor the legacy of any precipitation treatment. Accordingly, data from the altered precipitation plots were binned with data from the ambient plots and data from the warming + altered precipitation treatments were binned with data from the warming treatments allowing us to look explicitly at the effects of warming on *B. tectorum* phenology. Warming did not affect the timing of *B. tectorum* germination in any of the experiments. In contrast, both flowering and senescence significantly advanced with warming in all 3 experiments (Figure 2 and Table 2). For the CV4 and CV2 experiments, of all the phenophases analyzed, warming had the largest effects on the number of vegetative days, followed by flowering phenology, and lastly the timing of senescence (Table 2). These patterns shifted in the M2 experiment, which showed the largest warming effects on vegetative days, followed by senescence, then flowering (Table 2). Despite no significant germination responses to warming treatments, our models for each experiment suggest that, across years, mean germination dates were delayed in warmed plots relative to controls (Figures 3G,H,I and Table 2). These small and non-significant delays in conjunction with large significant warming-induced advances in flowering led to decreases in vegetative days representing the largest treatment effects (Figure 3 and Table 2).

When estimating the effects of interannual variation on the warming treatments, the conditional modes of the random effects showed that, in the earlier years (2010 and 2011), flowering and senescence were not consistently different between warming and ambient treatments, but flowering and senescence consistently advanced after 2014. With only one year of Fall germination recorded in the early years, it is difficult to compare earlier and later year patterns, but modeled conditional modes of the random effects show mixed earlier and later germination timing with respect to the means, and no obvious directional trend over time (Figures 3G,H,I).

Linear mixed-effects models examining the relationships between mean annual microclimate (10 cm soil moisture and temperature) and *B. tectorum* phenological timing only found a significant relationship between soil moisture and the timing of senescence in the CV4 experiment. In the CV4 experiment, senescence was estimated to occur 1.06 days earlier with every 1% increase in mean annual volumetric water content. All other relationships between soil moisture and phenological timing were not significant. On the other hand, all but two phenophases were significantly related to the mean annual soil temperatures. Warmer temperatures were associated with significant advances in the timing of flowering by 2.98–3.32 days per degree Celsius increase. Senescence also significantly advanced with warmer temperatures as an increase of 1 °C led to an advance of 1.28, 0.75, and 3.51 days in the CV4, CV2, and M2 experiments, respectively. Additionally, the vegetative growing season (time from germination to flowering) was significantly related to soil temperatures: warmer temperatures led to shorter growing seasons by 4.76–5.82 days per degree Celsius increase. Germination was the only phenophase that was delayed in warmer conditions and was estimated to occur 1.27 days later per degree Celsius increase. Marginal r^2^ values were <0.1 for all phenophases at all sites except for flowering where marginal r^2^ values were 0.21, 0.16, and 0.17 in the CV4, CV2, and M2 experiments, respectively. Conditional r^2^ values were always greater than 0.65 (Table 3).

**TABLE 3:** Results from mixed effects models analyzing the effects of 10 cm soil moisture and temperature on the timing of *B. tectorum* germination, flowering, senescence, and length of vegetative growing season.

Site	Phenophase	Mean annual soil moisture parameter estimate (DOY/%VWC)	Mean annual soil moisture standard error	Mean annual soil temperature parameter estimate (DOY/°C)	Mean annual soil temperature standard error	Marginal r^2^	Conditional r^2^
CV4	Germination	0.71	1.24	**1.27**	0.52	0.01	0.87
CV2	Germination	–0.21	0.81	1.79	0.94	0.01	0.84
M2	Germination	–0.11	0.99	1.99	1.4	0.01	0.89
CV4	Flowering	–1.11	0.8	**−3.13**	0.36	0.21	0.75
CV2	Flowering	–0.89	0.55	**−2.98**	0.6	0.16	0.66
M2	Flowering	–0.22	0.4	**−3.32**	0.8	0.17	0.73
CV4	Senescence	**−1.06**	0.48	**−1.28**	0.21	0.04	0.92
CV2	Senescence	–0.29	0.25	**−0.75**	0.29	0.01	0.95
M2	Senescence	–0.65	0.55	**−3.51**	1.1	0.06	0.84
CV4	Vegetative growing season	–2.14	1.62	**−4.76**	0.68	0.09	0.88
CV2	Vegetative growing season	–0.64	1.01	**−4.87**	1.17	0.06	0.85
M2	Vegetative growing season	0.54	1.32	**−5.82**	1.87	0.08	0.86

*Model parameters show the relationship between 10 cm soil moisture and soil temperature in units of DOY/%VWC and DOY/°C, respectively. DOY is the Julian day of year and %VWC is the percent volumetric water content of soils at 10 cm depths in units of cm^3^ H_2_O / cm^3^ soil. When model parameters are negative increases in moisture or temperature correspond to earlier phenology, when parameters are positive increases in moisture or temperature correspond to later phenology. Significance of the relationship between either soil moisture or temperature and *B. tectorum* phenology timing are determined with Type-II ANOVA and model parameters are italicized when *p* values < 0.1 and > 0.05 or bolded when *p* values < 0.05. Marginal and conditional r^2^ values were determined with the r.squaredGLMM function in the ‘car’ package (Nakagawa and Schielzeth, 2013; Fox and Weisberg, 2019). Marginal r^2^ values show how much variation in phenology timing can be explained by the additive effects of 10 cm soil moisture and soil temperature. Conditional r^2^ values show how much variation in phenology timing is explained by other aspects of interannual variation and by spatial variation across plots.*

## Discussion

We predicted that warming would advance *B. tectorum’s* phenological stages and that altered precipitation treatments would cause phenological delays. We found some support for our warming predictions: although we saw no significant warming treatment effects on germination, warming did significantly advance *B. tectorum* flowering and senescence, resulting in a shortening of the warmed plants’ vegetative growing season (Figure 3 and Table 2). In contrast, we did not detect any effects of the altered precipitation treatments on *B. tectorum* phenology. This is most likely due to the fact that altered precipitation treatments occurred outside of the *B. tectorum* growing season and that these treatments were only ongoing during two years of this study. Non-etheless, we did not observe changes in *B. tectorum* phenology in response to altered precipitation treatments or the legacy of those treatments.

In tracking *B. tectorum* phenology for seven years across a decade in multiple climate manipulation experiments, we found warming treatments had no significant effect on the timing of germination. This may be partially explained by research showing that the timing of germination is genetically restricted and cannot be explained by interannual precipitation and temperature variation, although the rate of germination is controlled by environmental conditions (Beckstead et al., 1996). However, while overall treatment effects on germination were not significant and varied among years, germination was the only phenophase to be delayed rather than advanced in the warming plots, and this was true for each experiment. The relationship between mean annual soil temperature and germination corroborates these treatment effects as warmer years were associated with later germination events (Table 3). *Bromus tectorum* seeds have been shown to shift thermal optimums for germination with changing levels of soil moisture: at low levels of soil moisture emergence rates are decreased by higher temperatures, but at higher levels of soil moisture emergence rates are increased by higher temperatures (Meyer and Allen, 1997). This speaks to the interactive effects of soil moisture and temperature on germination and lends insight into why warming treatments had larger effects in some years than others.

In contrast to germination, both flowering and senescence were significantly advanced by the warming treatments in all three climate manipulation experiments. On average, *B. tectorum* flowering in the +4 °C warmed plots occurred ∼8 days earlier than for plants in the unwarmed plots, whereas senescence occurred ∼3 days earlier. For both flowering and senescence, we observed significant effects of the degree of warming, with larger effects with higher warming levels (e.g., the 4 °C warming in CV4 vs. the 2 °C warming in CV2) (Table 2). Thus, these data suggest that the amount of warming effects the magnitude of the phenological response even across only a 2–4 °C range. The patterns of advancing flowering and senescence under warmer conditions was supported by trends in the soil microclimate analysis across years, which showed that mean annual 10 cm soil temperature was significantly related to flowering and senescence timing at all sites and warmer years had earlier occurrence of these phenophases (Table 3). Soil moisture may be playing an important role in the timing of flowering and senescence phenology, but we did not observe this relationship in our analysis. Responses to warming may also be influenced by competitive dynamics with native species in our plots (e.g., Walther et al., 2009) and future work would benefit from exploring these interactions further.

The strongest effects of the warming treatment were seen for the total number of vegetative growing days, with 12.1 fewer days of vegetative growth with warming in the CV4 experiment, 9.2 fewer days in the CV2 experiment, and 6.3 fewer days in the M2 experiment. By advancing the timing of the later phenological stages (flowering and senescence) without a corresponding shift in the timing of germination, the vegetative growing season of *B. tectorum* was effectively shortened under warmer conditions. Here too the effects of warming scaled with the magnitude of the temperature treatment, with larger effects in the +4°C treatments compared with plant responses in the +2 °C treatment (Table 2). The trends of shorter vegetative growing seasons under warmer conditions were also seen in our soil microclimate analysis across years, with significant negative relationships between mean annual soil temperature and growing season length in all experiments (Table 3). Although *B. tectorum* can cope and persist with increased temperatures, a shorter growing season in warmer environments may be evidence of increased stress in a warmer world, which could reduce *B. tectorum* success relative to ambient conditions. For example, in a Great Basin Desert study, shorter vegetative growing seasons led to decreased seed production (Mack and Pyke, 1983). Additionally, in a global meta-analysis, herbaceous species that flowered earlier were shown to exhibit shorter heights, decreased seed sizes, shallower roots, and smaller less dense leaves, showing that earlier phenology is tied with metrics that determine a plant’s success (Wolkovich and Cleland, 2014). Future research elucidating the interactive controls of phenology, growing season length, plant fitness, and seed production, as well as explorations of competitive interactions between *B. tectorum* and native plants under altered climate, would improve predictions of climate change’s effect on this common invasive grass. In light of *B. tectorum’s* large negative effects on invaded ecosystems (Young et al., 1987; Melgoza and Nowak, 1991; Anderson and Inouye, 2001; Humphrey and Schupp, 2001; Svejcar and Sheley, 2001) and of the substantial resources used attempting to control the invasive plant, an improved understanding of how and where *B. tectorum* will be affected by climate change would be of great value.

Contrary to the strong effects of warming, the altered precipitation treatments in our experiments did not significantly affect any of *B. tectorum’s* phenological stages in the years the experimental watering occurred (2009–2012) or in the years following. Additionally, in the microclimate analysis, mean annual 10 cm soil moisture was only significantly related to one phenophase in one experiment (senescence in CV4; Table 2). Determining how precipitation affects phenology is difficult, in part due to the strong interactions between temperature and soil moisture (Cleland et al., 2007). However, greenhouse experiments have shown no effect of watering amount on phenological differences for multiple source populations of *B. tectorum*, except for a delay in senescence (Rice et al., 1992). Conversely, the timing of water application has been shown to have strong effects on *B. tectorum* phenology, with winter moisture being particularly important (Prevéy and Seastedt, 2014). With this in mind, the lack of altered precipitation treatment effects in our study may have been due to the fact that the treatment was designed to ask questions about the monsoon and thus water was applied in summer months, which are effectively outside the *B. tectorum* growing season. Additionally, altered precipitation treatments occurred only in two of the years used for this analysis and therefore the analysis is better suited to examine legacy altered precipitation effects, and no legacy effects were seen. It is also possible that, although water is clearly a dominant driver of numerous ecological patterns in water-limited drylands, temperature may exert larger controls over *B. tectorum’s* phenology. Invasive plant phenology may be more strongly tied to temperature in systems where precipitation is variable, sporadic, and difficult to predict (Marushia et al., 2012; Winkler et al., 2018).

In addition to asking questions about how *in situ* climate manipulation affected the phenology of this invasive grass, the longer-term nature of the experiment also allowed us to ask questions about how phenology varied with interannual variations in background climate. We observed substantial interannual variation in the timing of all four measured phenological stages. Interestingly, while germination phenology showed little response to experimental warming, there were large differences in germination timing across years. For example, differences in the timing of germination initiation among years varied by up to 40 and 94 days in the CV experiments and the M2 experiment, respectively. In comparison, the maximum differences in germination timing between warmed and ambient plots within a year were 11 days at CV4 and 30.6 days at M2. In other words, the largest effects of experimental warming were small relative to the range in timing seen across years for *B. tectorum* germination. We observed similar patterns in the effects of experimental warming vs. interannual variation for senescence and flowering. Annual plants are particularly plastic in their response to interannual variation in temperature and precipitation compared to perennials (Wainwright et al., 2012). The patterns we observed across years and with treatments align with other studies suggesting that phenotypic plasticity in *B. tectorum* may make the plant particularly adept at dealing with extreme interannual climate variation, except in the cases where insufficient winter moisture is available (Funk, 2008; Wainwright et al., 2012; Prevéy and Seastedt, 2014). Indeed, studies suggest *B. tectorum* senesces prior to the driest summer conditions as a drought avoidance strategy that allows the species to persist in arid environments (Thill et al., 1984; Rice and Mack, 1991). *Bromus tectorum’s* ability to alter senescence, and the timing of flowering, shows a flexibility that could help the species closely track changing climatic conditions. Further, global data suggest plant species that flower earliest in the growing season have the highest phenological temperature sensitivities, thus, part of the strong temperature responses we observed in flowering and senescence may be due to *B. tectorum* life history traits (Sherry et al., 2007; Wolkovich et al., 2012; Wolkovich and Cleland, 2014). Accordingly, although the shortened growing season with warming treatments and in warmer years may point to higher plant stress as temperatures rise, the large phenological plasticity observed for *B. tectorum* could also suggest success for the invasive plant in a world where climate is changing.

In exploring drivers of *B. tectorum* phenological plasticity across years, we found soil temperature to be significantly associated with the timing of germination, flowering, and senescence, and with the length of the vegetative growing season. Mean annual soil temperature was significantly related to the timing of all phenophases except germination in M2. In contrast, mean annual soil moisture was only significantly linked to senescence timing in CV4 (Table 3). Additionally, the marginal r^2^ values showed that mean annual soil moisture and temperature do not explain a large portion of the variation in timing of all phenophases, suggesting additional controls. Here it is important to note that by estimating soil temperature and moisture at the annual scale, we are almost certainly missing cues and events that play an important role in promoting phenological advance. This is particularly important in the context of seasonal rain events that would not be captured well by an annual average. For instance, multiple studies have shown that the timing of germination is tied with large precipitation events, and that late season precipitation can delay senescence (Sakai et al., 2001; Winkler et al., 2018). Soil moisture is likely a component driving phenological responses in our experiments, but this relationship was difficult to detect, in part because for most of the year the soils in these dryland experiments were dry. However, while the experimental warming effects on soil moisture did not lead to significant deviations from ambient soil (Figure 1), there is the likelihood that warming-induced soil drying could contribute to the warming effects on *B. tectorum* phenology and previous research at the CV site showed that warming dried soils at 5 cm depths in both the CV4 and CV2 experiments during 2010 and 2011 (Wertin et al., 2015). Nevertheless, more work elucidating climate controls on and predictions for finer-scale patterns of *B. tectorum* phenology would improve forecasts of how future temperature and precipitation patterns will alter *B. tectorum* phenology.

**FIGURE 4:**
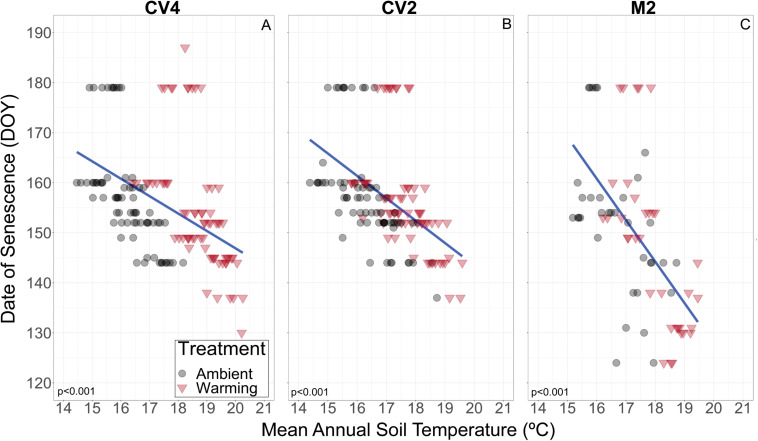
Plot level relationship of date of *B. tectorum* senescence and mean annual soil temperature at 10 cm depth in the CV4, CV2, and M2 experiments (**A–C**, respectively). Values from warmed and ambient plots are shown with red triangles and black circles respectively. The linear fits are represented by blue lines and were determined by the geom_smooth function in ggplot2. The linear fits incorporate data from all plots (Ambient and Warming) in each site. R^2^ values for each linear fit is shown in the bottom left of each panel.

We also observed differences across the two sites in the responses of *B. tectorum* phenology to experimental warming and annual microclimate variation. When comparing the +2 °C warming effects in the CV2 experiment with the +2 °C warming effects in the M2 experiment, we found that the flowering advances with warming were nearly 1.5 times higher in the CV2 experiment, while advances in senescence were five times greater in the M2 experiment (5 day warming treatment-induced advance in M2 vs. a 1 day advance in CV2). Similarly, senescence in the M2 experiment was more sensitive to natural inter-annual temperature fluctuations than senescence in the CV2 experiment: a 1°C increase was related to a 3.5 day advance in M2 and only a 1.28 and 0.75 day advance in CV4 and CV2, respectively (Figure 4). This suggests different sites will vary in not only the magnitude of their *B. tectorum* phenology response to warming, but also in the degree to which specific aspects of phenology are affected (i.e., warming had a larger effect on flowering phenology in CV2 than M2, but a smaller effect on senescence). Soil texture may help explain the between-site variation in the controls of soil moisture and temperature. The limited water-holding capacity of coarse, sandy soils, such as those found at the M2 site, often drive higher variability in soil moisture and may lead to greater variability in soil temperature between rain events (Santos et al., 2019). It has been suggested that *B. tectorum* abundance and cover are higher in finer-textured soils compared to more coarse, sandy soils (Belnap et al., 2016) and the increased variation of soil moisture in sandy soils may help explain this preference. A better understanding of how *B. tectorum* phenology responds to changing temperatures across multiple sites and texture gradients is necessary for predicting the future success of the invasive plant.

Although our data point to a shortened *B. tectorum* growing season with both 2 and 4°C increases in temperature, the large phenological flexibility we observed suggest warmer conditions could also afford *B. tectorum* a competitive advantage over the perennial plants it lives amongst if those perennial plants are not able to respond to changing climate as quickly. We did not analyze phenological responses to warming of co-occurring species here, however, previous research in these experiments has shown a dominant native perennial species has experienced dramatic declines in cover in response to warming, potentially enabling *B. tectorum* to take advantage of this newly opened space and further invade the site (Winkler et al., 2019). Due to the large effect of *B. tectorum* on native perennials, any climate change effects on the invasive grass could, in turn, affect the success of native plants (Willis et al., 2010). For example, *B. tectorum* winter growth has been shown to inhibit germination success of co-located native perennials (Harris, 1977; Aguirre and Johnson, 1991). *Bromus tectorum’s* roots also develop rapidly after fall germination, and as temperatures cool in the winter the plant becomes semi-dormant (Klemmedson and Smith, 1964; Aguirre and Johnson, 1991). If increased warming is sufficient to cancel dormancy effects of colder winter temperatures, warming could allow *B. tectorum* to stay metabolically active, and continue root growth, permitting increased exploitation of resources before natives are active (Ashbacher and Cleland, 2015). Although we did not find advances in the timing of fall germination with experimental warming, the substantial advance of flowering suggests that *B. tectorum* shifted its life stages earlier in response to increased temperatures. Such a shift would be expected to include the uptake of limited soil resources, and thus these changes could affect plant competition. In all, the large flexibility we observed in *B. tectorum* with warming and across years suggests a strong potential to track climate in way that may support the plant’s success even in the face of climate change.

Large changes in the timing of *B. tectorum* life stages represent management challenges that additional data could help address. In particular, understanding the fate of this invasive grass with future climate would be of significant use to land managers, as would specific predictions of how phenology will change across time. Management of *B. tectorum* often focuses on spraying or grazing the plant prior to it setting seed (Whitson and Koch, 1998; Lehnhoff et al., 2019). If increasing temperatures create both earlier seed set and increased variability of the timing of seed set, this would create additional logistical challenges for determining when and where to treat *B. tectorum* across the landscape. The unique climate manipulation experiments presented here provide insight into the mechanisms through which this highly successful invasive plant may respond to changes in climate, and the effects of experimental warming on *B. tectorum* phenology offer a glimpse into how the common invasive grass will respond to predicted future climate conditions. The reduced vegetative growing season, the high plasticity observed, and the differences seen across sites improve our understanding of *B. tectorum’s* ability to closely track climatic conditions, which provides forecasting power for the timing of key life stages. Overall, the findings from these experiments can inform predictions of when germination, flowering, and senescence may occur in order to more effectively manage invaded areas and to help identify which ecosystems may be prone to invasion with the continued effects of climate change.

## Data Availability Statement

The raw data supporting the conclusions of this article will be made available by the authors, without undue reservation.

## Author Contributions

AH synthesized and collected the data and wrote the first draft of the manuscript. AH, BM, and MP led the statistical analyses of the data. DW contributed a broader global change perspective to data interpretation. SR acquired funding and provided project leadership. All authors contributed to the idea development, data interpretation, and the writing of the manuscript.

## Conflict of Interest

The authors declare that the research was conducted in the absence of any commercial or financial relationships that could be construed as a potential conflict of interest.
