# Supplementary material for: Experimental Warming Changes Phenology and Shortens Growing Season of the Dominant Invasive Plant Bromus tectorum (Cheatgrass)
Source: Front Plant Sci. 2020 Oct 15;11:570001. doi: 10.3389/fpls.2020.570001 (PMC7593257; doi:10.3389/fpls.2020.570001)
Supplement: Supplementary file 1 [file Data_Sheet_1.PDF]

**Perennial**	**Annual**
Achnatherum hymenoides (Roemer & J.A. Schultes) Barkworth	*Bromus tectorum* L.
Allium spp.	*Cryptantha tenuis* (Eastw.) Payson
Aristida purpurea Nutt.	*Cymopterus acaulis* (Pursh) Raf.
Astragalus amphioxys A. Gray	*Descurainia pinnata* (Walter) Britton
Atriplex confertifolia (Torr. & Frem.) S. Watson	*Eriastrum spp.*
Chaetopappas ericoides (Torr.) G.L. Nesom	*Erodium cicutarium* (L.) L'Hér. ex Aiton
Chamaesyce fendleri Torr. & A. Gray	*Gilia inconspicua* (Sm.) Sweet
Delphinium andersonii A. Gray	*Gilia spp.*
Encelia frutescens A. Gray	*Ipomopsis polycladon* (Torr.) V.E. Grant
Ephedra viridis.	*Lappula spp.*
Eriogonum inflatum Torr. & Frem.	*Lepidium montanum* Nutt.
Gaillardia pinnatifida Torr.	*Malcolmia Africana* (L.) W.T. Aiton
Gutierrezia sarothrae (Pursh Britton & Rusby)	*Phacelia crenulata* Torr. ex S. Watson
Hesperostipa comata (Trin. & Rupr.) Barkworth	*Plantago patagonica* Jacq.
Heterotheca villosa (Pursh)	*Salsola tragus* L.
Lygodesmia grandiflora (Nutt.) Torr. & A. Gray	*Stephanomeria exigua* Nutt.
Machaeranthera canescens (Pursh) A. Gray	*Streptanthella longirostris* (S. Watson) Rydb.
Mirabilis linearis (Pursh) Heimerl	*Vulpia octoflora* (Walter)
Oenothera caespitosa Nutt.	
Opuntia spp.	
Pleuraphis jamesii Torr.	
Sclerocactus whipplei Engelm. & J.M. Bigelow	
Sphaeralcea parvifolia A. Nelson	
Sporobolus contractus Hitchc	
Stanleya pinnata (Pursh) Britton	
Tiquilia latior (I.M. Johnst.) A.T. Richardson	

Supplemental Table 1. Experiment level species list for CV4 divided into annual and perennial species. This list includes all species that were found within the plots.

**Perennial**	**Annual**
Achnatherum hymenoides (Roemer & J.A. Schultes) Barkworth	*Bromus tectorum* L.
Allium spp.	*Chaenactis stevioides* Hook. & Arn.
Astragalus amphioxys A. Gray	*Cryptantha tenuis* (Eastw.) Payson
Atriplex confertifolia (Torr. & Frem.) S. Watson	*Cymopterus acaulis* (Pursh) Raf.
Chaetopappas ericoides (Torr.) G.L. Nesom	*Descurainia pinnata* (Walter) Britton
Chamaesyce fendleri Torr. & A. Gray	*Erodium cicutarium* (L.) L'Hér. ex Aiton
Delphinium andersonii A. Gray	*Festuca octoflora* Walter
Ephedra viridis.	*Gilia inconspicua* (Sm.) Sweet
Eriogonum inflatum Torr. & Frem.	*Gilia spp.*
Gaillardia pinnatifida Torr.	*Ipomopsis polycladon* (Torr.) V.E. Grant
Gutierrezia sarothrae (Pursh Britton & Rusby)	*Lappula spp.*
Hesperostipa comata (Trin. & Rupr.) Barkworth	*Lepidium montanum* Nutt.
Krascheninnikovia lanata (Pursh) A. Meeuse & Smit	*Malcolmia Africana* (L.) W.T. Aiton
Lygodesmia grandiflora (Nutt.) Torr. & A. Gray	*Phacelia crenulata* Torr. ex S. Watson
Machaeranthera canescens (Pursh) A. Gray	*Plantago patagonica* Jacq.
Oenothera caespitosa Nutt.	*Salsola tragus* L.
Opuntia spp.	*Stephanomeria exigua* Nutt.
Pleuraphis jamesii Torr.	*Streptanthella longirostris* (S. Watson) Rydb.
Sclerocactus whipplei Engelm. & J.M. Bigelow	*Townsendia incana*Nutt.
Sporobolus contractus Hitchc	
Tiquilia latior (I.M. Johnst.) A.T. Richardson	

Supplemental Table 2. Experiment level species list for CV2 divided into annual and perennial species. This list includes all species that were found within the plots.

Perennial	Annual
Achnatherum hymenoides (Roemer & J.A. Schultes) Barkworth	*Ambrosia acanthicarpa* Hook.
Agropyron cristatum (L.) Gaertn.	*Bromus tectorum* L.
Artemisia filifolia Torr.	*Cryptantha tenuis* (Eastw.) Payson
Atriplex canescens (Pursh) Nutt.	*Cymopterus acaulis* (Pursh) Raf.
Coleogyne ramosissima Torr.	*Descurainia pinnata* (Walter) Britton
Ephedra viridis Coville	*Erodium cicutarium* (L.) L'Hér. ex Aiton
Eriogonum inflatum Torr. & Frem.	*Helianthus petiolari*s Nutt.
Machaeranthera canescens (Pursh) A. Gray	*Lepidium montanum* Nutt.
Mentzelia multicaulis (Osterh.) A. Nelson ex J. Darl.	*Lupin spp.*
Oenothera caespitosa Nutt.	*Malcolmia Africana* (L.) W.T. Aiton
Oenothera pallida Lindl.	*Phacelia ivesiana* Torr.
Opuntia spp.	*Plantago patagonica* Jacq.
Sporobolus contractus Hitchc	*Salsola tragus* L.
	Sisymbrium altissimum L.
	Sphaeralcea parvifolia A. Nelson
	Stephanomeria exigua Nutt.
	Streptanthella longirostris (S. Watson) Rydb.
	Vulpia octoflora (Walter)

Supplemental Table 3. Experiment level species list for M2 divided into annual and perennial species. This list includes all species that were found within the plots.

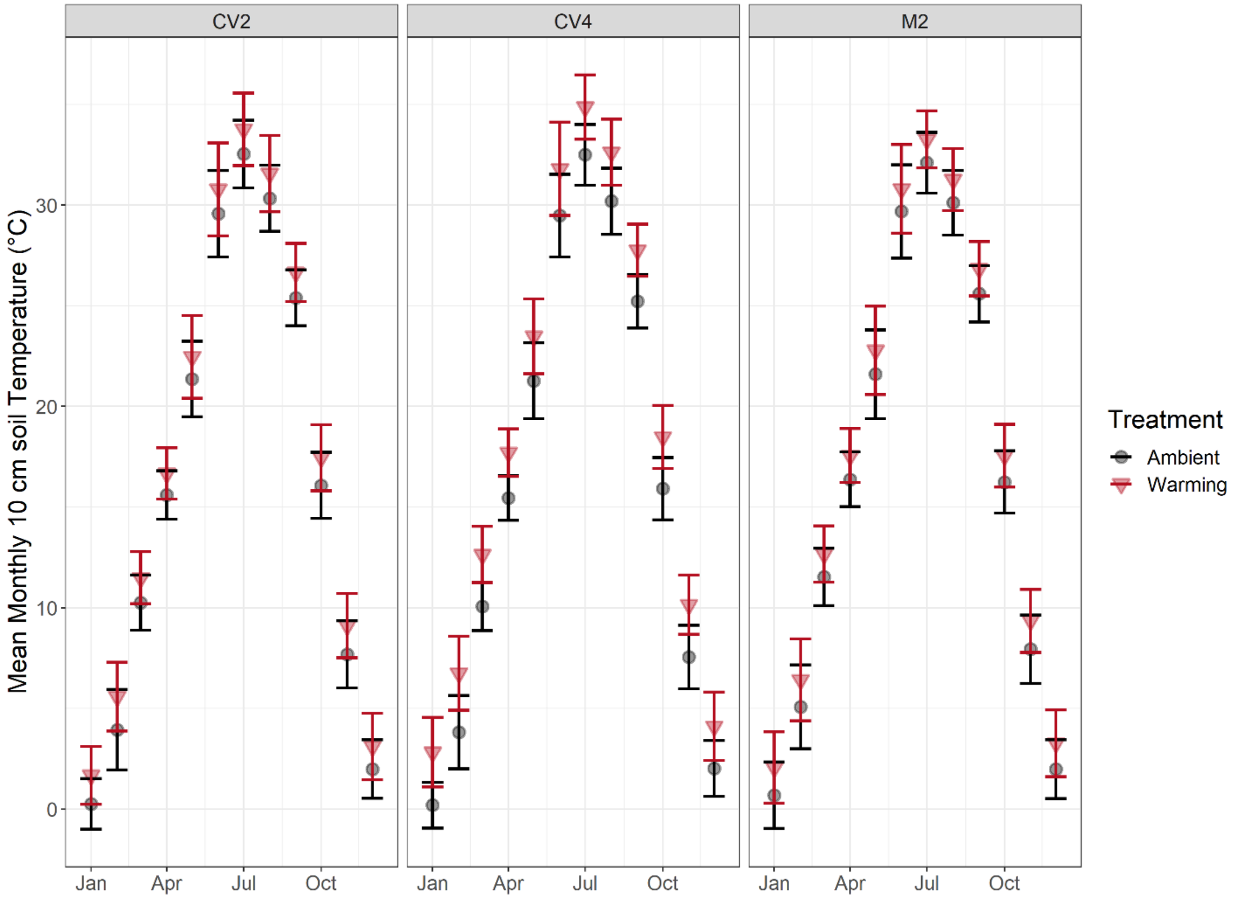

Supplemental Figure 1. Mean monthly 10 cm soil temperatures in each experiment. Mean monthly temperature values were determined across all years from 2009-2019. Error bars represent standard deviations for each month.
